# Gaps and opportunities in addressing the needs of older adults in the Philippines and Vietnam: a qualitative exploration of health and social workers’ experiences in urban care settings

**DOI:** 10.3389/fpubh.2024.1269116

**Published:** 2024-03-22

**Authors:** TJ Robinson T. Moncatar, Man Thi Hue Vo, Kathryn Lizbeth L. Siongco, Tran Dai Tri Han, Kaoruko Seino, Aliya Vanessa D. Gomez, Carmelita C. Canila, Richard S. Javier, Thang Van Vo, Yuri Tashiro, Fely Marilyn E. Lorenzo, Keiko Nakamura

**Affiliations:** ^1^Department of Health Policy and Administration, College of Public Health, University of the Philippines Manila, Manila, Philippines; ^2^College of Public Health Sciences, Chulalongkorn University, Bangkok, Thailand; ^3^College of Nursing, University of the Philippines Manila, Manila, Philippines; ^4^Faculty of Public Health, Hue University of Medicine and Pharmacy, Hue University, Hue, Vietnam; ^5^World Health Organization Collaborating Centre for Healthy Cities and Urban Policy Research, Tokyo, Japan; ^6^Department of Global Health Entrepreneurship, Division of Public Health, Tokyo Medical and Dental University, Tokyo, Japan; ^7^College of Public Health, University of the Philippines Manila, Manila, Philippines; ^8^Institute for Community Health Research, Hue University of Medicine and Pharmacy, Hue, Vietnam; ^9^Nurses Christian Foundation, Manila, Philippines

**Keywords:** older adults, geriatric care, unmet needs, urban health, qualitative research, Philippines, Vietnam

## Abstract

**Background:**

Despite numerous government initiatives, concerns and disparities among older adults have continually been growing. Empirical studies focused on older adults in the Philippines and Vietnam appear minimal and mostly regarding perceptions of aging. An effective geriatric care strongly relies on functional service providers requiring their perspectives to be explored toward inclusive service delivery.

**Objective:**

To investigate the perceived gaps and opportunities in geriatric care service delivery among health and social care workers in selected urban areas in the Philippines and Vietnam.

**Methods:**

A qualitative case study approach drawn on social constructivism theory, examined working experiences, observed characteristics of older adults, geriatric services and needs, difficulties on service delivery, and recommended solutions. A total of 12 semi-structured interviews and 29 focus group discussions were conducted in the Philippines, with 174 health and social care workers, while in Vietnam, there were 23 semi-structured interviews and 29 focus group discussions with 124 participants. An inductive thematic analysis was employed.

**Results:**

Interview participants highlighted the increasing unmet needs such as accessibility, availability, and acceptability of geriatric care services. The implementation of interventions on the older population faced multiple challenges, including issues related to older adult conundrums and dilemmas in geriatric care providers and facilities. The participants from the two countries felt that strengthening implementation of collaboration toward an integrated geriatric care structure and expansion of training and capability in handling older adults can be potential in addressing the gaps at both individual and institutional levels. Additionally, a committed leadership was viewed to be the important step to effectively operationalize the strategy.

**Conclusion:**

Health and social workers emphasized that the needs of older adults are exacerbated by various challenges within a fragmented geriatric care system. To address this issue, an establishment of an integrated service delivery mechanism with dedicated leadership is needed. The findings from this study may help develop appropriate solutions for addressing the health and social care needs of older adults in similar settings across Southeast Asia. Further examination of the impact of these challenges and solutions on service delivery and the wellbeing of older adults is essential.

## 1 Introduction

The population in South-East Asia is rapidly aging at a significant pace. According to the United Nations, 12.1% of the population in the region were aged 60 years or over in 2022 (1). This demographic shift is primarily due to increased life expectancy and declining fertility rates, which bring significant challenges for health care, social security, and economic growth within the region. As aging progressing swiftly in the region, the more developed countries have been the pioneers in transitioning into an aging society. For instance, as of 2022, Thailand has become the second most aged society in the region, with people 65 years and above accounting for more than 14 percent of the total population (2), following the footsteps of Singapore in 2019 (3).

Despite the acceleration of aging currently being more evident in the high and upper-income countries in the region, it is crucial to tackle the aging issue in lower-middle-income countries as well. In countries like Philippines and Vietnam, although benefiting from the optimal population structure with the working-age population comprising more than half of the total at present (4, 5), there is a risk that these countries could experience significant social and economic impacts when this rapidly ongoing demographic transition is underway. By 2050, it is estimated that there will be 34 countries worldwide with at least 10 million older adults, and both Vietnam and the Philippines are expected to join the list (6). Subsequently, the health and social care needs of the aging population are becoming more complex and demanding. In less developed countries, it is challenging to distribute healthcare resources efficiently due to the economics of aging and care financing, requiring consideration of various factors such as healthcare financing and delivery systems, social security, and long-term care, while the awareness of the issues related to the older population is low (7). For countries that have not traditionally had to deal with the issue of aging, there would also be a need for a change in perspective on the part of healthcare professionals (8).

Both classified as lower middle-income countries and undergoing a demographic shift toward aging populations, Vietnam and the Philippine also share cultures that traditionally place a strong emphasis on community and family support (9, 10). They also encounter challenges in providing sufficient healthcare services for their aging populations. The governments from the two countries have been making efforts to develop a long-term care system to cater for their older population (11). Some policy framework toward geriatric care on older people have been in place in Vietnam (12), or laws and programs focusing on services for older Filipinos are continuously developed in the Philippines (13). Nevertheless, many major issues remained, such as the lack of inclusiveness to accommodate the poor Filipinos, inadequate provision of basic health and social care needs (14), inequitable access to care services (15), or lack of training resources for geriatric specialties in Vietnam provincial hospitals (16). Additionally, there are distinct differences that may give rise to differing challenges and opportunities in geriatric care among the two countries. In the Philippines, a critical challenge comes from the local scarcity of healthcare workers, especially as the demand for Filipino nurses keeps growing in other developed countries, leaving the country with a lack of experienced workforce (17). In the case of Vietnam, an ongoing issue is the unequal utilization of and insufficient financial protection for the older population within the context of the current social health insurance scheme (18).

As the Philippine and Vietnam’s populations age, it will be important to have more time for investment in health and social care for the older population. Despite the differing aging structures and variation in health and social care access and quality in the Philippines and Vietnam, our focus is on uncovering any similarities or extreme differences in of the two countries on the aging issue, and explore whether health and social care workers from the two countries perceive differences in geriatric care service delivery for their growing aging population. The comparison may provide insight into national and regional geriatric issues by allowing academics and policymakers to see how countries with diverse backgrounds address geriatric concerns. As information on the gaps and opportunities that affect older adults’ access to geriatric care service delivery is important for developing comprehensive health strategies, the findings are expected to aid in developing informed decision-making, improved healthcare services, and consequently enhanced well-being for the aging populations in both countries. Furthermore, this may potentially serves as a case study for other countries within South-East Asia that face comparable geriatric challenges. Also, to our knowledge, there is a paucity of studies on the perceived gap and opportunities in geriatric care delivery in Vietnam and the Philippines, especially in relation to the early time of implementation of various laws and regulations, and when the accessibility of healthcare resources are mostly prominent in urban areas or big cities (16, 19, 20). Therefore, this study aims to explore the perceived gaps and opportunities in geriatric care service delivery among health and social care workers from various health care settings in urban areas in the Philippines and Vietnam.

## 2 Materials and methods

### 2.1 Research design

This qualitative study utilized a case study approach (21) anchored on social constructivism theory that asserts the knowledge and understanding of an individual is mainly shaped by their interactions within their social context (22). This ensured that the shared knowledge and experiences of individual interview participants are due to their group social dynamics fostering the scientific epistemology of the study. This facilitated the in-depth interviews or focus group discussions (FGDs) (ranging from two to 15 participants) among health and social care workers from various health care settings who are tasked with preventive, curative, and rehabilitative interventions, and welfare and support services for older adults to explore the gaps and opportunities in geriatric care delivery in the Philippines and Vietnam. The utilization of FGD with homogenous groupings of health and social care workers per health facility was the main method of qualitative inquiry in this study as it aims to assess mutual agreements and disagreements, ensure comfort, openness, and higher degree of interaction among the participants. Consequently, in-depth interview was also utilized due to uncontrolled circumstances (i.e., only one available participant or working in a specific facility during schedule of inquiry), but enabled the team to further discuss and probe on sensitive issues that may be difficult to obtain or missed during FGDs of similar profession.

### 2.2 Study settings

Two cities in the Greater Manila Area (GMA), Philippines and one province in Central Vietnam were purposely selected due to its increasing number of older adults and urban area. These study sites also have several types of facilities such as tertiary hospitals, nursing homes, primary care centers (i.e., community health centers or stations), and religious centers (i.e., church, temples) directly addressing health and social care needs of older adults. In particular, in the Philippines, one public and three private tertiary hospitals, two city health units, four community health centers, and four nursing homes participated in the study. In Vietnam, four public hospitals, 20 community health centers, one nursing home, and two Buddhist temples were selected for data collection.

### 2.3 Sampling and recruitment

The research team received a formal approval from local government officials to approach health and social workers in various care settings to take part in the study. Staff from the local government also identified and suggested hospitals and nursing homes that offer care to older adults for inclusion. After giving both written and verbal explanations regarding the study, consent was then requested. The study’s description was disseminated to employees who met the inclusion criteria—being a caregiver for older individuals and currently working as a health or social worker. After potential study participants gave their agreement, only then were interviews and FGDs conducted. The study sample consisted of every health and care worker available during the data collection period. Numerous professionals involved in the implementation of programs and services for older individuals, including doctors, nurses, physical therapists, and social workers, were questioned to determine the extent of the gaps in geriatric care services. Additionally, non-professionals with training in their fields were also included in this study, including community health workers, dietitians, and community leaders tasked with linking older adults to various health and social services. We also spoke with caregivers, nuns, and staff in charge of helping older adult residents of nursing homes with their activities of daily living (ADL). In addition, other relevant offices actively providing geriatric care services were subsequently identified by government officials and were snowballed into the study. Specifically, 174 health and social workers participated in 12 in-depth interviews and 29 focus group discussions in the Philippines, whereas 124 health and social workers participated in 23 FGDs in Vietnam.

### 2.4 Data collection and procedure

The in-depth interviews and FGDs using a semi-structured guide covering observed characteristics of older adults, experiences related to gaps between patient needs and services rendered, and difficulties of older adults in accessing health and social welfare services were primarily conducted by two researchers per country who took turns in facilitating and note-taking. To ensure privacy and confidentiality, interviews were done in rooms with only the researchers and participants present. With the participants’ approval, all interviews were audio recorded and lasted between 45 to 90 min. Three researchers supervised the collection of all the data to ensure quality and robustness.

### 2.5 Data analysis

With assistance from trained research assistants, interviews were verbatim transcribed and translated into English. In order to identify questions to further explore during following interviews, analysis started during data collection. To ensure agreement, the transcripts were compared against field interview notes, reflective memos, and discussions between senior research members. NVivo 12® (QSR International, Burlington, MA, United States) was used to handle the data, and an inductive thematic analysis approach was applied to help identify the descriptive codes following Braun and Clarke’s six-phase guide (23). Reading the transcriptions frequently, creating and fine-tuning the codes, and looking for initial themes were all part of phases one through three. The initial theme was developed using preliminary codes that were pertinent to the study subject. These themes were examined and defined in phases four and five. Report writing was required at the last stage. From phases two to six of the analysis, there was continuous code and theme processing and improvement. All transcripts were subjected to this procedure repeatedly until analytical saturation was reached (24). Senior research team members also examined and validated the themes, presented the analysis to a small group of interviewees and important stakeholders for comment, and made sure that the analysis process was rigorous (25). Narrative quotes were selected to present the key themes and to ensure the transparency of shared perceptions strengthening the trustworthiness of our analysis.

### 2.6 Ethical considerations

This study received approval from four Ethics Review Board: the World Health Organization Research Ethics Review Committee [ERC.0003093], Tokyo Medical and Dental University Medical Research Ethics Committee [M2017-232], the Philippine Department of Health Single Joint Research Ethics Board [SJREB-2018-21], and Hue University of medicine and Pharmacy [H2018/148 12 May 2018]. Written informed consent forms, study information, and a chance to withdraw participation or ask questions were provided to all study participants. The research team rigorously adhered to participant names and responses’ anonymity and confidentiality.

## 3 Results

Table 1 showcases the themes which emerged from the collected data, highlighting the gaps in caring for older adults and their corresponding solutions, as perceived by health and social care workers at the primary care level. Challenges identified by the informants encompass the burden of caring for older adults, zeroing in on their conundrums, and dilemmas from the end of providers and facilities. Zooming further in, said conundrums subsume the unmet needs and observed disparities in care access; while the latter constitutes resource barriers, providers’ frustrations, and the fragmentation of the care system for older adults.

**Table 1:** Challenges in caring for older adults in the Philippines and Vietnam, and perceived solutions by health and social care workers at the primary care.

Themes	Sub-Themes
Older adult conundrums	Increased unmet needs Disparities in health and social care access
Geriatric care provider and facility dilemma	Exacerbated resource constraints Apparent struggles and frustrations of care providers Fragmented geriatric care system
Resolutions to the predicaments in caring for older adults	Committed leadership and prioritization of geriatric care Expansion of training and capability in handling older adults Strengthened collaboration to advance integrated geriatric care system

Correspondingly, solutions deemed by the care professionals to be crucial in addressing the gaps entail leaders’ commitment to and prioritization of geriatric care, training, capacity-building activities, and more robust collaboration to promote the care system integration for geriatric patients.

### 3.1 Older adult conundrums

One of the gaps in geriatric care identified by the interviewed health and social care workers encompasses the adversities experienced by the older population within Vietnam and the Philippines, contributing to geriatric care as a constant difficulty.

#### 3.1.1 Increased unmet needs

First and foremost, hindrances experienced by older adult people zero in on the observed rise in their unmet needs, compounded by the incongruence when it comes to their care access.

Primarily, informants in both countries shared that there had been a rise in the need for health and social care for the older adult population, mainly due to the increased incidence of various diseases, ranging from communicable and non-communicable to functional cognitive and sensory difficulties. Physicians who took part in the discussions in the Philippine and Vietnam also claimed that older adults consult with them almost every week, complaining of symptoms that they fear are indicators of serious illnesses. Community health workers also noted a rise in older adult patients needing wheelchairs, and walkers, and generally, are bed-ridden.

The rate of stroke among older patients is increasing rapidly, with patients ranging from 60 to 70 years old, and even 80 to 90 years old. In the past, patients above the age of 90 didn't usually go to the hospital, but there are now some cases where they do seek medical attention. (Doctor, University Hospital in Vietnam, FGD)

In the Philippines, informants observed vaccine hesitancy among their older adult population, perceived to be caused by a vaccination fiasco in the country several years ago.

Vaccinations for flu and pneumonia are also affected. We should have been administering it every year. There really is a stigma with regards to vaccination nowadays. If only the patients are well-informed, we wouldn’t have been having scattered incidences of measles. Our senior citizens become scared as well, making them hesitant to be vaccinated, unlike before when they are the ones asking for vaccine shots. (Nurses and Rehabilitation Therapists, Primary Care Center in the Philippines, FGD)

#### 3.1.2 Disparities in health and social care access

The FGD informants in both countries also pinpointed the apparent and prevalent issue of inequities in access to health and social care, thereby affecting the overall health status and quality of life of older adult people. Owing to a number of socioeconomic factors, the care providers included that source of income, low economic status, and geographical access difficulties comprise these. Other patients were also observed to be heavily reliant on pensions, or on their spouses for their respective medical expenditures. With all these barriers combined, older adults belonging to vulnerable populations continue to experience difficulty in accessing the services they require, consequently putting them at increased health and social risks.

Majority of the inpatients are very poor… Patients who suffer from stroke, and injuries normally have to pay a lot in other hospitals before moving here, so they become poorer. Secondly, the trends in some diseases are increasing among the younger population, unlike before. In the past, stroke was common among patients aged 60 and above, but now this was found in younger patients, from 45 years old. (Doctor, Rehabilitation Hospital in Vietnam, FGD)

There are senior citizens who find it hard to travel to healthcare facilities. I hope there are facilities near them. (Social Worker, Public Hospital in the Philippines, FGD)

Those from the poorest households do not receive assistance as they are not informed. Those who do mainly have connections with the officials. (Physician, Primary Care Center in the Philippines, FGD)

People who used to work for the government are in better health and social condition compared to the ones who are not. They (informal retired workers) are poorer in both physical and mental aspects. (Doctor, Primary Care Center in Vietnam, FGD)

### 3.2 Geriatric care provider and facility dilemma

Supplementarily, barriers within facilities and among care providers were also agreed upon as a contributor to the experienced burden in service delivery for older adult people. Such revolve around the worsened resource barriers, disappointments of the care professionals, and the overall fragmentation of the care system for geriatric patients.

#### 3.2.1 Exacerbated resource constraints

The informants from both the Philippines and Vietnam highlighted constraints revolving around equipment shortage, substandard facilities, lack of human resources, insufficient budget, and a meager salary. The complaints about deficiencies in equipment and facilities do not just cause inconvenience to the patients, but the providers as well. Additionally, insufficiency in the budget translates to the experienced medicine stock-outs, the low salary among providers, and struggles with program implementation; while the shortage in the workforce results in insufficient time spent with patients, which affects service availability as well.

If the hospital has sufficient facilities, the staff can feel reassured when treating patients. Staff members can quickly upgrade their skills within one or two years. While human recruitment may present some challenges, it should not be a major issue. However, patient rooms, equipment, and facilities are difficult to change once they are built and may require a decade before upgrades are feasible. (Doctor, University Hospital in Vietnam, FGD)

Older adults in the last stage of disease or in the last stage of life have a lot of care needs. But the doctors do not have enough time to help them. (Doctor, Primary Health Center in Vietnam, FGD)

We have a difficult profession, since you exert so much effort physically, especially for those bedridden patients. It would have been fine if they were all ambulatory patients. But if they are bedridden, we use a lot of our physical strength and health. So, for me, we don’t get paid enough. (Caregiver, Nursing Home in the Philippines, FGD)

These resource constraints also manifest through the limited health coverage of the national health insurance program, as noted by participants in the Philippines, making it hard for older adult patients to settle medical bills.

I hope they’ll fix the health financing and the insurance policy. They should expand the packages and the coverage for senior citizens. (Nurse, Primary Health Care Center in the Philippines, FGD)

#### 3.2.2 Apparent struggles and frustrations of care providers

Participants also enumerated a number of difficulties they experience, encompassing hardships in managing older adults, and in collaborating with other professionals. Specifically, Filipino and Vietnamese health and social care workers shared that they experienced older adults patients being hardheaded, and show a negative attitude toward them. Others developed unhealthy lifestyle practices like consumption of excessive sodium, compounded by minimal water intake. If continued, these can lead to more serious health conditions.

Patients coming here really want to be consulted about nutrition. For example, hypertensive patients should eat less salt, but they ate the soy sauce as an alternative and assumed that it is less salty than other sauces. Their knowledge about nutrition is limited. Doctors advise consuming less salty foods, but they don’t understand. (Doctor, Public Hospital in Vietnam, FGD)

Vietnamese participants also claimed that cultural background can be a barrier to service utilization among adults. Geriatric patients’ perspective on social clubs affects their engagement in the activities.

They (Older adults) do not prioritize social activities. They have to spend their time staying home to take care of their grandchildren, or do other jobs, even run their own business, because of their family situations. (Staff, Primary Health Care Center in Vietnam, FGD)

#### 3.2.3 Fragmented geriatric care system

Overall, the Filipino and Vietnamese health and social care workers who were part of the FGDs asserted that the current care system for geriatric patients remains to have significant gaps — lack of care options and services, coordination, a streamlined array of services between various settings, monitoring of services, communication among staff, and capacity-building activities for care providers, among others.

At this hospital, it is obvious that we don’t have that kind of service (Home care service). It is such a regret for patients who have those needs. There is no service on nursing, feeding, and common problems. (Doctor, University Hospital in Vietnam, FGD)

We aren’t properly diagnosing those with Alzheimer’s and dementia, so we really need to focus on awareness building and orientation in terms of handling geriatric patients. (Nurse, Public Hospital in the Philippines, FGD)

In the Philippines, it was shared that there is little to no standard when it comes to service delivery, contributing to the subpar coordination among providers.

I hope there is a standard procedure we can follow. Right now, we’re all on our own, there’s no coordination. (Nurse, Public Hospital in the Philippines, FGD)

It’s hard to provide care if you do not have complete resources and guidelines to follow. (Nurse, Public Hospital in the Philippines, FGD)

On the other hand, informants from Vietnam shared viable options like home care services as potential solutions to these identified challenges. Such services would benefit patients requiring help with their daily activities.

Currently, in Vietnam, services are quickly developing but home care services and facilities for the semi-acute stage are still not yet implemented. (Doctor, University Hospital in Vietnam, FGD)

### 3.3 Resolutions to the predicaments in caring for older adults

Corresponding to the identified gaps by the informants, solutions entailing leaders’ involvement, prioritization, capacity building, and collaboration were also suggested.

#### 3.3.1 Committed leadership and prioritization of geriatric care

Prioritization of geriatric care among leaders was believed to pave way for a more improved array and coverage of services. Furthermore, such commitment to geriatric health will aid in filling the gaps in the health and social care needs of the older adult population, thereby advancing the overall quality of geriatric care.

If only funds and budget are allocated to the national level for the senior citizens, we would be more efficient in providing services. (Community Health Worker, Primary Health Care Center in the Philippines, FGD)

We need additional funds since with budget increase, others will follow — social welfare, medicines, and other needs of older adult people. We just need finances. (Community Health Worker, Primary Health Care Center in the Philippines, FGD)

There was also a consensus among participants in Vietnam that a model for geriatric care will aid patients in efficiently navigating the service delivery system from the acute phase to long-term care.

Geriatric care involves acute, semi-acute, and chronic stages. Acute care is provided in hospitals, and there is a need to improve the quality of hospital rooms. Semi-acute care focuses on patients' recovery and exercise, and there is a high demand for this type of care. Home care services and regular monitoring are needed for patients who are at home. (Doctor, University Hospital in Vietnam, FGD)

I want a model that can follow up with patients even after their discharge. That would provide the best care quality for patients. To be honest, there are patients who are not completely recovered when they return home, but because of their economic situation, they have to leave the hospital. After that, we don’t see them anymore, so our effort treating them in the hospital becomes meaningless. (Rehabilitation Therapist, Public Hospital in Vietnam, FGD)

#### 3.3.2 Expansion of training and capability in handling older adults

Supplemental training courses for care providers are also believed by Filipino and Vietnamese participants to be necessary and shall be based on specific training needs between various professions, and care settings. For one, participants from the Philippines emphasized the need for greater awareness among professionals about their role as service providers, enabling improvements in the service delivery network.

In terms of the referral system, we have a lot of senior citizens who do not receive the care they need due to a lack of awareness of those around them, may it be family members or doctors. They do not know how to refer the cases to us; so, we’re just here in the hospital, while they’re admitted to the ward. There is no proper education as to where [in the course of treatment] we come in, when in fact, we should have been with the patient right from their admission to the hospital. All these [are experienced] despite the presence of health care professionals, what more if the patient is in the community? There’s a large gap when it comes to awareness and education. (Rehabilitation Therapist, Public Hospital in the Philippines, FGD)

The informants also believed that the capacity of family members in providing care for older adults can be enhanced through similar training activities.

The care quality for older adults from their family members is not good… I think there should be some training or activity to enhance the awareness of home care knowledge provided for the main caregiver of older adults. (Physical therapist, University Hospital in Vietnam, FGD)

#### 3.3.3 Strengthened collaboration to advance integrated geriatric care system

The participants from both countries believed that handling and caring for older adult people also entailed a robust collaboration among healthcare professionals, so as to further advance service delivery. They expressed that it is essential that care providers avoid competition, and instead, build positive relationships, forge collaboration, and practice effective communication. Such cooperation between professionals will pave way for the needed continuity of interventions, thereby benefiting the patients.

You surely need partners who will continue, so that it’s not just us who are responsible for the patient’s recovery, as we are not the only ones addressing their problems. Our patients have several problems, ranging from physical to mental. Everything. That’s why we need a holistic approach. (Rehabilitation Therapist, Public Hospital in the Philippines, FGD)

It is better if we have teamwork skills. Everyone finds a better approach to patient care. (Rehabilitation Therapist, Public Hospital in Vietnam, FGD)

Collaboration is also believed to be essential between different professions, and with local government offices.

We need to have case conferences, with the city and municipal social welfare and development offices, so that we can be aware if they can help address the needs and problems of our senior citizens. (Social Worker, Public Hospital in the Philippines, FGD)

For us, it is convenient because we have professors who are both doctors and lecturers of the nursing department at the medical university. The professor will be the coordinator in the briefings, two or three times per week. Hence in the briefing, the nurses can learn a lot. (Nurse, University Hospital in Vietnam, FGD)

## 4 Discussion

Although aged care facilities have been gradually established in the Philippines and Vietnam to enhance the care of older patients, the lack of scientific evaluations raises uncertainty about whether these interventions adequately meet the needs of older patients, particularly from the perspective of care providers. Despite the geographical differences and varying stages of the aging process, the provision of geriatric care was perceived to present specific challenges to healthcare and social workers in both countries. Nevertheless, these professionals also see some solutions to the difficulties they face in caring for older adults.

Unmet needs, coupled with disparities in accessing adequate care were seen as major conundrums faced by older patients. These challenges appeared to be uniform in both countries. In Vietnam, disparities in health and social care access, especially among vulnerable groups, demand targeted interventions and increased healthcare investment. Meanwhile, in the Philippines, financial insecurity among older Filipinos necessitates comprehensive policies and social support systems to ensure their economic security and life quality. Regardless of the variation in disparity and unmet need between the two countries, the fundamental challenge remains consistent. Our results are consistent with recent reports on geriatric care in both countries, where their Universal Health Coverage (UHC) indexes - the key indicator measuring service quality, coverage, and financial protection (26), have yet to reach their maximum potential (Vietnam: 70, Philippines: 55) (27). Furthermore, it has been indicated that countries with higher UHC Service Coverage Indexes typically have a lower prevalence of unmet health need (28). Financial constraints could be the primary cause for the unmet need, since one out of every five older individuals in the Philippines had an unmet for health care due to this reason (13), which is similar to the situation in Vietnam where not having enough money is one of the most frequent reasons why older patients do not seek care (29). The unmet demand for healthcare and social care also highlights disparities in care, as exemplified by the role of financial stability for older adults, particularly those who were previously part of the informal workforce in both countries. In the Philippines, financial insecurity for older adults is intensified by the prevalence of informal employment, which makes it challenging for them to save for their retirement, and the government’s social pension program is only available to indigent senior citizens (30). In Vietnam, a non-contributory social pension scheme was introduced to cover formal pensioners and to attract more workers from the informal sector (31), However, this system has been struggling to keep up with the aging population and progress has been slow. This is mainly because the program is highly focused on the top 40% of income earners, the poorest workers, and those aged 80 and above, leaving a significant coverage gap for individuals aged between 60 and 79 years old (32). Furthermore, gender inequality remains a concern, as fewer women are able to access social insurance pensions (33). Another potential explanation for the unmet needs and care access disparity among older adults in both the Philippines and Vietnam is their shared lack of awareness (29) and utilization (13) of their rights and the government’s programs that are available to support them.

One of the common challenges that arise in the dilemma faced by geriatric care providers and care facilities is the exacerbation of resource constraints. The scarcity of financial and human resources is a persistent problem in countries such as the Philippines and Vietnam in the Asia-Pacific region, and it is not a new challenge (6, 34). Inadequate healthcare services in the Philippines were attributed to staffing, drug supply, and health center accessibility issues (30). Similarly, Vietnam faced a lack of funding to implement healthcare policies and insufficient long-term care personnel (35). Policy makers, along with relevant stakeholders, should take into account the identified needs when allocating budget and funding to geriatric care services.

Health and social care workers also identified challenges in managing and collaborating with older patients. While older patients may need specialized care that considers their unique physical, emotional, and cultural needs, the availability of health and social care workforce with specialized knowledge and training in the two countries remains an issue. For instance, in the Philippines, Catholicism reinforces caregiving as a family responsibility, and other community services may be stigmatized, causing families to decline or use them secretly (36). As well, in agreement with participant’s concern about older patients’ dietary habit, excessive salt intake in Vietnam, surpassing WHO recommendations (37), remains a significant issue intricately tied to the daily diet and proves challenging to address or modify as a lifestyle risk factor (38). These can create challenges in providing care for older patients with diverse cultural backgrounds. Managing older patients may require a patient-centered approach that considers their unique needs, requiring skilled and resourceful professionals.

The participants expressed concern that the geriatric care system in the Philippines and Vietnam is insufficient and has not kept up with the demands of providing comprehensive and integrated care for older adults. The lack of robust coordination and fragmented services are not the challenges for only the two countries, but also for other countries in the region (6), although there has been an increasing recognition of the need to accelerate the development of geriatric care system in recent years. Due to the tradition in both the Philippines and Vietnam of relying on family members with little external support to provide geriatric care services (35, 39), the government may have a lesser responsibility for providing such care, exemplified by the lack of institutional care services for older adults (40). Another reason why homes for the aged are rare is that the cost of such services is higher than the average income of older adults, which is the case in Vietnam (29). Also, it has been reported that the benefits offered by health insurance to the majority of older Filipinos are still insufficient to cover the full cost of healthcare (13), and the accessibility of some resources and benefits was reported to be dependent on the health service providers (41). In response to a Vietnamese participant’s raised concern about geriatric service system, there has been indeed a growing recognition to enhance adherence to hospital practice guidelines in low-and middle-income countries like Vietnam as a means to mitigate adverse outcomes following patient discharge (42). Indeed, it is true that the formal support structures for the aging population in the two countries are still in the process of being developed (13), requiring medium and long-term policies (35).

Despite all the challenges faced, participants agree that addressing the challenges of geriatric care requires committed leadership and prioritization of geriatric care, expanding training and capabilities in managing older adults, and strengthening collaboration. All of this aligns with policy recommendations proposed for the development of a geriatric care system in both the Philippines and Vietnam (34, 35, 43), aimed at tackling various challenges, including inadequate awareness of the aging issue, leadership, and resource allocation. For instance, to utilize informal caregivers for older adults effectively, short-term training can be done to enhance the caring skills of informal home-based caregivers. The Technical Education and Skills Development Authority could potentially provide these trainings in the Philippines to assist caregivers in managing the mental, emotional, and physical strain of their role (13). Similar to other countries with aging populations in Asia, this solution reveals a lack of collaboration among different sectors and disciplines when it comes to providing care for older adults (44). Collaboration is also considered as critical in geriatric care as it requires a multi-faceted approach (45) involving diverse professionals, organizations, and community resources working together to meet the distinctive needs of older adults. Our results also agreed with commonly identified competencies, including communication, role clarity, and teamwork, within the framework of essential capabilities required for a health professional to practice (46). To facilitate this, the commitment and agreement among pertinent stakeholders and geriatric programs are pivotal. This involves active participation and collaboration among health and social care providers, geriatric care institutions, governmental agencies overseeing geriatric and gerontology services, community organizations dedicated to supporting programs for older individuals, and advocacy groups focused on enhancing the well-being of older adults. This multifaceted engagement ensures a comprehensive and unified approach to address the complex challenges associated with geriatric care. It is noteworthy that recently, both Vietnam (47) and the Philippines (48, 49) have been placing emphasis on the significance of collaboration and the development of interprofessional education programs.

It is important to consider the strengths and limitations of this study. The first strength is that it is the first qualitative study exploring the perspective of care providers in geriatric care in both Vietnam and the Philippines. Another strong point of this study is the extensive participation of various healthcare and social service providers with diverse professional backgrounds. However, the study’s results may not be representative of rural areas, and data collection was conducted in 2019, which may not reflect the current situation. Additionally, it is important to note that this study did not consider the perspectives of the care recipients. Moreover, the diversity in terms of regional variations and socioeconomic disparities within the two countries may necessitate more meticulous considerations in sample selection in future studies to ensure a better representation of their entire populations. Despite these limitations, the findings of this study emphasize the need for policymakers and public health workers in the two countries to take action toward strengthening the geriatric care system as their population continues to age quickly.

## 5 Conclusion

This study identified that health and social workers from selected health care settings working with older adults in the Philippines and Vietnam perceived that a convergence of factors, primarily older adults conundrums and geriatric care provider and facility dilemma, worsen the unmet needs of older adults. Therefore, it was construed that institutionalization of an integrated geriatric service delivery system based on committed leadership and governance and training in handling older adults must be strengthened to address the predicaments in caring for older adults. Furthermore, it is necessary to conduct additional research on how these issues and their resolutions affect older adults’ wellbeing and service delivery.

## Data availability statement

The raw data supporting the conclusions of this article will be made available by the authors upon request.

## Ethics statement

The studies involving humans were approved by World Health Organization Research Ethics Review Committee [ERC.0003093], Tokyo Medical and Dental University Medical Research Ethics Committee [M2017-232], the Philippine Department of Health Single Joint Research Ethics Board [SJREB-2018-21], and Hue University of medicine and Pharmacy [H2018/148 12 May 2018]. The studies were conducted in accordance with the local legislation and institutional requirements. The participants provided their written informed consent to participate in this study.

## Author contributions

TM: Funding acquisition, Investigation, Methodology, Project administration, Resources, Software, Supervision, Validation, Visualization, Writing – original draft, Writing – review & editing, Conceptualization, Data curation, Formal analysis. MV: Conceptualization, Data curation, Formal analysis, Investigation, Methodology, Project administration, Software, Supervision, Validation, Visualization, Writing – original draft, Writing – review & editing. KLS: Funding acquisition, Investigation, Resources, Supervision, Validation, Writing – review & editing. TH: Data curation, Investigation, Project administration, Supervision, Validation, Visualization, Writing – review & editing. KS: Funding acquisition, Project administration, Resources, Software, Supervision, Validation, Writing – review & editing. AG: Formal analysis, Visualization, Writing – original draft, Writing – review & editing. CC: Project administration, Resources, Supervision, Writing – review & editing. RJ: Project administration, Supervision, Validation, Writing – review & editing. VT: Funding acquisition, Project administration, Resources, Supervision, Validation, Writing – review & editing. YT: Project administration, Resources, Validation, Writing – review & editing. FL: Conceptualization, Formal analysis, Methodology, Project administration, Supervision, Validation, Writing – review & editing. KN: Conceptualization, Formal analysis, Funding acquisition, Methodology, Project administration, Resources, Software, Supervision, Validation, Writing – review & editing.
